# Role of the CYP3A4-mediated 11,12-epoxyeicosatrienoic acid pathway in the development of tamoxifen-resistant breast cancer

**DOI:** 10.18632/oncotarget.20329

**Published:** 2017-08-18

**Authors:** Nguyen Thi Thuy Phuong, Ji Won Kim, Jung-Ae Kim, Jang Su Jeon, Ji-Yoon Lee, Wen Jun Xu, Jin Won Yang, Sang Kyum Kim, Keon Wook Kang

**Affiliations:** ^1^ College of Pharmacy and Research Institute of Pharmaceutical Sciences, Seoul National University, Seoul 08826, South Korea; ^2^ College of Pharmacy, Yeungnam University, Gyeongsan 38541, South Korea; ^3^ College of Pharmacy, Chungnam National University, Daejeon 34134, South Korea

**Keywords:** tamoxifen resistance, CYP3A4, epoxyeicosatrienoic acid, migration, angiogenesis

## Abstract

Epoxyeicosatrienoic acid (EET) production via cytochrome P450 (CYP) epoxygenases closely correlates with the progression of breast cancer. However, its role in the development of chemoresistant breast cancers has yet to be elucidated. Here, we found that CYP3A4 expression and its epoxy-product, 11,12-epoxyeicosatrienoic acid (11,12-EET) was enhanced in tamoxifen (TAM)-resistant MCF-7 (TAMR-MCF-7) breast cancer cells compared to control MCF-7 cells. Treatment of TAMR-MCF-7 cells with ketoconazole and azamulin (selective CYP3A4 inhibitors) or 14,15-epoxyeicosa-5(Z)-enoic acid (14,15-EEZE, an EET antagonist) inhibited cellular proliferation and recovered the sensitivity to 4-hydroxytamoxifen. Chick chorioallantoic membrane and trans-well migration analyses revealed that the enhanced angiogenic, tumorigenic, and migration intensities of TAMR-MCF-7 cells were also significantly suppressed by ketoconazole and 14,15-EEZE. We previously reported that Pin1, a peptidyl prolyl isomerase, is a crucial regulator for higher angiogenesis and epithelial-mesenchymal transition characteristics of TAMR-MCF-7 cells. EET inhibition suppressed E2F1-dependent Pin1 gene transcription, and Pin1 silencing also blocked cell proliferation, angiogenesis, and migration of TAMR-MCF-7 cells. Our findings suggest that the CYP3A4-mediated EET pathway represents a potential therapeutic target for the treatment of tamoxifen-resistant breast cancer.

## INTRODUCTION

Approximately 2 of 3 breast cancers require estrogen and functional estrogen receptor α (ER-α) for growth; thus, anti-estrogens such as tamoxifen (TAM), a non-steroidal anti-estrogen, have been widely used and have been the most effective therapy in the treatment of ER-positive breast cancer patients [1]. Although TAM treatment prolongs overall survival and reduces mortality and metastatic disease, most patients ultimately acquire resistance, which has been a serious problem in the treatment of breast cancer patients [2, 3]. However, the molecular mechanism leading to endocrine resistance and the poor prognosis of breast cancers remain controversial. Hence, the identification of an appropriate target is needed to contribute to the treatment of these endocrine-resistant breast cancers.

Cytochrome P450s (CYPs) are a complex group of enzymes that are required for the metabolism of diverse xenobiotics, including therapeutic agents and environmental procarcinogens [4, 5]. The expression of P450s correlates with both the response to chemotherapy and the initiation or promotion of tumorigenesis [4, 5]. One of the physiological functions of CYP epoxygenases is metabolizing arachidonic acid (AA) to biologically active eicosanoids termed cis-epoxyeicosatrienoic acids (EETs), including 5,6-EET, 8,9-EET, 11,12-EET, and 14,15-EET [6]. EETs are quickly metabolized, mainly by soluble epoxy hydrolase (sEH), to less-active dihydroxyeicosatrienoic acids [7]. EETs are well-known in the cardiovascular system, where they function as vascular relaxation factors independent of nitric oxide and prostacyclin I2 [8]. Moreover, EETs also exert the ability to stimulate angiogenesis with both secreted EETs and synthetic EETs [9–11]. In humans, CYP epoxygenases, including CYP2C8, 2C9, 2J2, and CYP3A4, have been well-known to convert AA to EETs [12–14]. The expression of these CYP epoxygenases has been reported to play a critical role in the tumorigenesis of diverse cancers, supporting a potential role for EETs in carcinogenesis. CYP2C8, CYP2C9, and CYP2J2 are overexpressed in three prostate carcinoma cell lines (PC3, DU-145, and LNCaP) and contribute to the metastatic capacity (invasion and migration) of these cell types [15]. Specifically, 11,12-EET is the major AA metabolite and potentiates cell invasion and migration in these prostate carcinoma cell lines [15]. CYP3A4 overexpression increases hepatocarcinoma growth, which can be inhibited by the addition of a putative EET-receptor antagonist, 14,15-epoxyeicosa-5(Z)-enoic acid (14,15-EEZE) [16]. CYP2J2-derived EETs have been shown to promote invasion and metastasis in a variety of human cancers, including breast carcinoma [17]. Mitra *et al.* demonstrated that CYP3A4 epoxygenase promotes the growth of estrogen receptor (ER)-positive breast cancer cells, in part through the biosynthesis of 14,15-EET [18]. Despite the increasing number of studies focusing on the roles of CYP epoxygenases and EETs in breast cancer, their effects on the development of TAM-resistant breast cancer have not yet been identified. The aim of this study was to identify the potential role of CYP epoxygenases and their derived EETs during the development of endocrine-resistant breast cancers.

Our research revealed that CYP3A4 is overexpressed and plays an important role in cell proliferation, angiogenesis, and migration in TAM-resistant breast cancer cells, in part through 11,12-EET biosynthesis. This finding suggests that inhibition of CYP3A4 and the EET signaling pathway may represent new therapeutic strategies for the treatment of endocrine-resistant breast cancers.

## RESULTS

### Expression of CYP epoxygenases and EET synthesis in TAMR-MCF-7 cells

**Figure 1:**
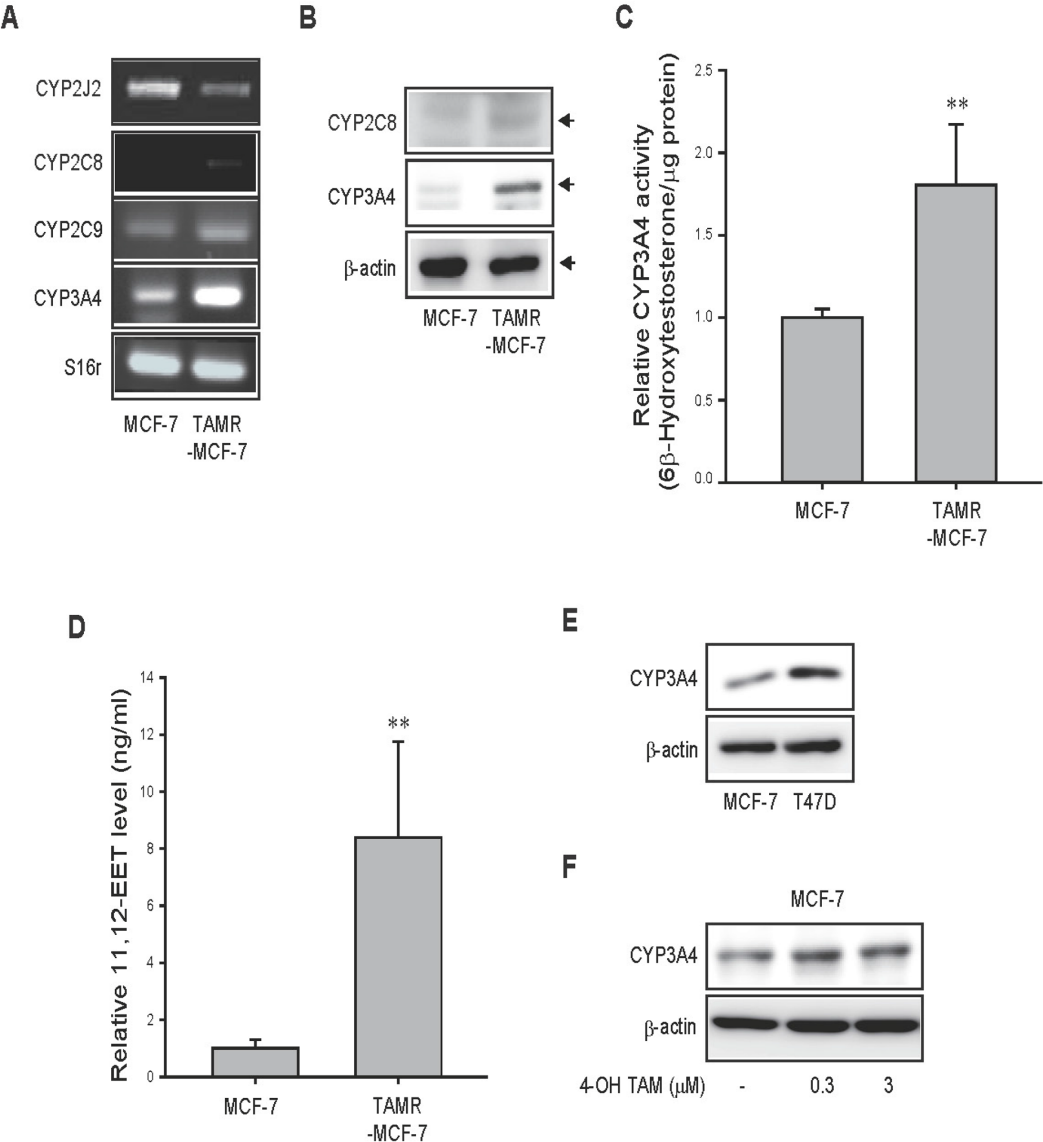
CYP epoxygenases expression and EETs level in MCF-7 and TAMR-MCF-7 cells **(A)** mRNA levels of CYP2J2, 2C8, 2C9 and 3A4 in MCF-7 and TAMR-MCF-7 cells. **(B)** Western blot analysis of CYP2C8 and CYP3A4 protein expression in MCF-7 and TAMR-MCF-7 cells. **(C)** CYP3A4 activity. MCF-7 and TAMR-MCF-7 cells were incubated with 200 μM testosterone for 6 h, and the amounts of 6β-hydroxytestosterone were determined. **(D)** 11,12-EET levels in MCF-7 and TAMR-MCF-7 cells. Extracted samples of both MCF-7 and TAMR-MCF-7 cells were submitted to LC-ESI/MRM/MS analysis in a mass chromatography coupled with HPLC assay and 11,12-EET product was determined. Data represent mean±SD with 4 different samples (significant versus MCF-7 cells, ^**^ P<0.01). **(E)** Comparison of CYP3A4 expression in MCF-7 and T47D cells. **(F)** Effect of 4-hydroxytamoxifen (4-OH TAM) on CYP3A4 expression. MCF-7 cells were exposed to 0.3 and 3 μM 4-hydroxytamoxifen for 24 h and the total cell lysates were subjected to CYP3A4 immunoblotting.

CYP epoxygenases, including CYP2C8, 2J2, 2C9, and CYP3A4, have the capacity to synthesize EETs and may be involved in breast cancer progression [18, 19]. We compared the mRNA expression levels of these epoxygenases in both MCF-7 and TAMR-MCF-7 cells. RT-PCR analysis revealed that the CYP3A4 mRNA level was dramatically increased in TAMR-MCF-7 cells compared to control MCF-7 cells, while CYP2C8 and CYP2C9 mRNA levels were only slightly enhanced, and the CYP2J2 mRNA level exhibited a decreasing trend (Figure 1A). Immunoblot analyses confirmed that the protein expression of CYP3A4 was clearly increased in TAMR-MCF-7 cells, and the levels of CYP2C8 and CYP2C9 were marginally changed (CYP2C8) or undetected (CYP2C9) according to cell type (Figure 1B). We then compared CYP3A4 enzyme activities between MCF-7 and TAMR-MCF-7 cells. After incubation of both the cell types with testosterone (CYP3A4 substrate), 6β-hydroxytestosterone formation was about 2-fold increased in TAMR-MCF-7 cells compared to MCF-7 cells (Figure 1C). Because CYP3A4 displays a high ability of AA epoxygenase in breast cancer [18], we next determined the levels of EETs in MCF-7 and TAMR-MCF-7 cells. Interestingly, 11,12-EET synthesis was selectively elevated approximately 8-fold in TAMR-MCF-7 cells compared to MCF-7 cells (Figure 1D), whereas 5,6-EET, 8,9-EET, and 14,15-EET were produced at a very low or undetectable concentrations in both the cell types (data not shown). These data suggest that 11,12-EET is the major epoxy metabolite of AA elevated in CYP3A4-overexpressing TAMR-MCF-7 cells. Although both MCF-7 and T47D cells are classified as luminal breast cancer cell lines, T47D cells are relatively more TAM-resistant clone [20, 21]. When we assessed protein level of CYP3A4, the basal expression levels of CYP3A4 in T47D cells was higher than those in MCF-7 cells (Figure 1E). Moreover, single exposure of 4-hydroxytomoxifen (0.3 and 3 μM) in MCF-7 cells marginally increased the protein expression of CYP3A4 (Figure 1F), which imply that CYP3A4 induction in TAM-resistant breast cancer cells may results from long-term adaption of cells to 4-hydroxytamoxifen.

### Role of CYP3A4-mediated EET production in cell proliferation and TAM-resistance in TAMR-MCF-7 cells

**Figure 2:**
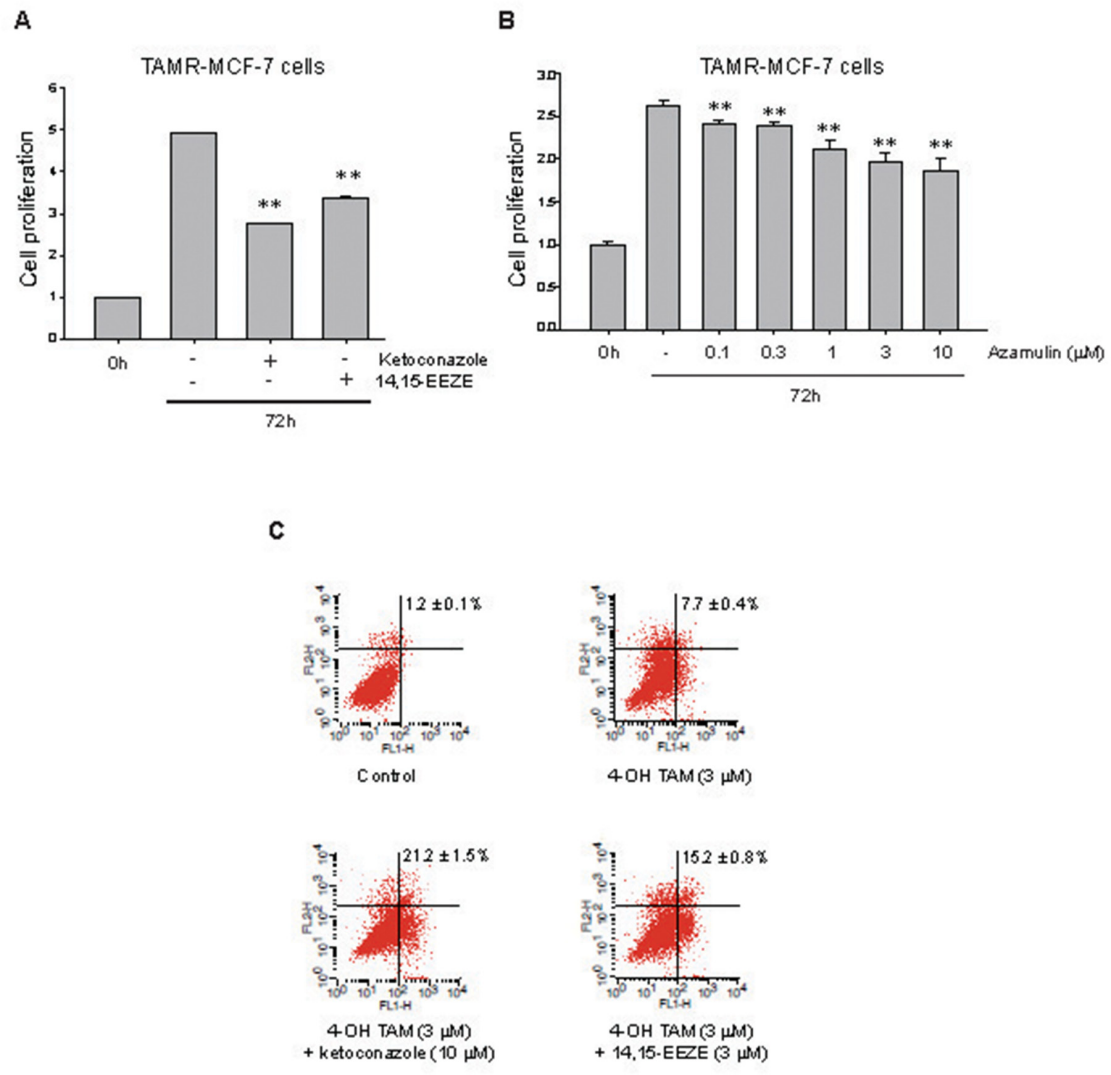
Effect of CYP3A4 inhibitors and 11,12-EET antagonist on cell proliferation and apoptosis in TAMR-MCF-7 cells **(A, B)** Effect of ketoconazole (A), 14,15-EEZE (A) or azamulin (B) on cell proliferation of TAMR-MCF-7 cells. TAMR-MCF-7 cells were plated in 96 well plate and cell proliferation rate was determined by MTT assays after 72 h treatment with ketoconazole (10 μM), azamulin (0.1-10 μM) and 14,15-EEZE (3 μM). Data represent mean±SD with 8 different samples (significant versus vehicle-treated TAMR-MCF-7 cells, **P<0.01). **(C)** Representative dot plots of TAMR-MCF-7 cells stained with annexin V-FITC (FL1-H) and propidium iodide (FL2-H) using flow cytometry assay. TAMR-MCF-7 cells were treated with 3 μM 4-hydroxytamoxifen (4-OH TAM) in the presence or absence of ketoconazole (10 μM) or 14,15-EEZE (3 μM) for 24 h. Lower right panel represents early apoptotic cells. Upper-left panel and upper-right panel represent late necrotic and apoptotic cells, respectively. Lower-left panel represents survival cells. The indicated percentage value is sum of apoptotic cells from lower right panel and upper-right panel (n=3).

It has been reported that CYP3A4 is expressed in approximately 80% of human breast cancers, and that enzyme expression correlates with decreased overall survival in breast cancer [22]. Additionally, EETs promote cell proliferation in several cancers including breast cancer [18]. To identify a potential role for CYP3A4-mediated EET production in TAM-resistant breast cancer cells, we used ketoconazole, azamulin (chemical CYP3A4 inhibitors) and 14,15-EEZE (an EET antagonist). We found that treatment with ketoconazole (10 μM), azamulin (10 μM) or 14,15-EEZE (3 μM) for 72 h significantly inhibited cell proliferation of TAMR-MCF7 cells by 51%, 41% and 40%, respectively (Figure 2A and 2B), suggesting that CYP3A4-mediated EET production partially contributes to the cellular proliferation of TAMR-MCF-7 cells. Moreover, flow cytometric analyses revealed that co-treatment of TAMR-MCF-7 cells with ketoconazole (10 μM) and 14,15-EEZE (3 μM) increased the number of annexin V-positive apoptotic cells compared to cells treated with 4-hydroxytamoxifen (3 μM) alone (Figure 2C). These data suggest that CYP3A4 overexpression is associated with the cellular proliferation and chemoresistance of TAMR-MCF-7 cells, in part through the enhanced biosynthesis of 11,12-EET.

### Role of CYP3A4-mediated EET production in VEGF-mediated angiogenesis of TAMR-MCF-7 cells

**Figure 3:**
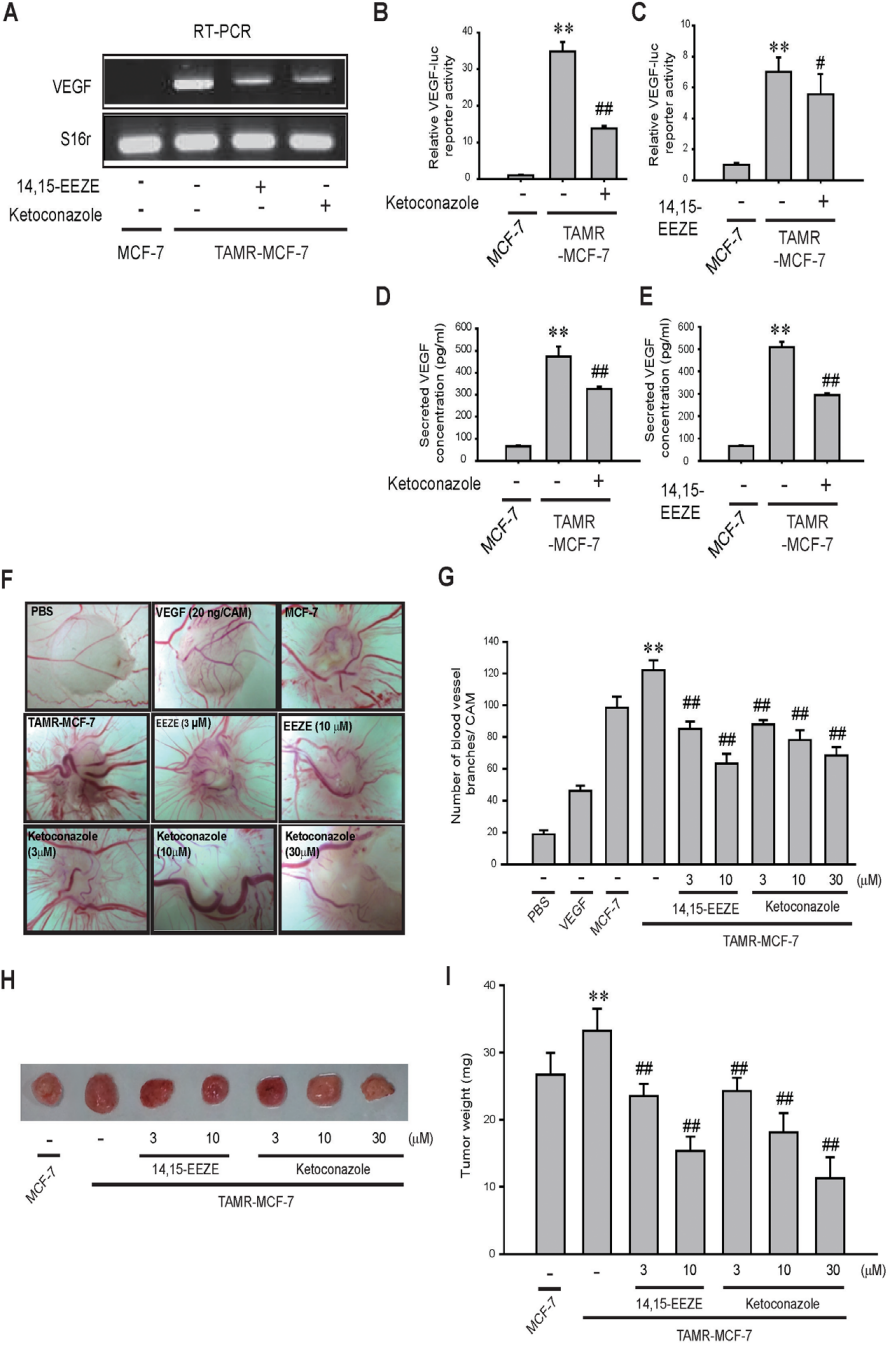
Effects of CYP3A4 inhibitor and EET antagonist on angiogenesis in TAMR-MCF-7 cells **(A)** Effect of CYP3A4 inhibitor or EET antagonist on VEGF mRNA level in TAMR-MCF-7 cells. TAMR-MCF-7 cells were exposed to ketoconazole (10 μM) and 14,15-EEZE (3 μM) for 24 h in serum free condition. VEGF mRNA level was determined by RT-PCR. **(B, C)** Effect of ketoconazole (10 μM) (B) and 14,15-EEZE (3 μM) (C) on the reporter gene activity of VEGF-luc in TAMR-MCF-7 cells. Each cell type was transiently co-transfected with VEGF-luc reporter plasmid (1 μg/ml) and phRL-SV (hRenilla) (1 ng/ml). Dual luciferase reporter assays were performed on the lysed cells 18 h after reagent treatment. Reporter gene activity was calculated as a relative ratio of firefly luciferase to hRenilla luciferase activity. Data represent mean±SD with 4 different samples (significant versus MCF-7 cells, ^**^P<0.01; significant versus vehicle treated TAMR-MCF-7 cells, ^##^ P<0.01). **(D, E)** Effect of CYP3A4 inhibitor and EET antagonist on secreted VEGF level. TAMR-MCF-7 cells were treated with ketoconazole (10 μM) (D) and 14,15-EEZE (3 μM) (E) for 24 h in serum deprived condition. Secreted VEGF concentration was measured by human VEGF ELISA kit. Data represent mean±SD with 3 different samples (significant versus MCF-7 cells, ^**^P<0.01; significant versus vehicle treated TAMR-MCF-7 cells, ^#^ P<0.05, ^##^P<0.01). **(F)** Representative pictures of angiogenesis originated from both MCF-7 and TAMR-MCF-7 cells using CAM assay. The control CAMs of a 10 day old chick embryo were exposed to PBS or VEGF (20 ng/ml). The additional embryos were implanted with MCF-7 or TAMR-MCF-7 (2x10^6^ cells/CAM). **(G)** The quantification of new small branches formed from existing blood vessels was performed 3 days after cancer cell implantation. Data represent mean±SD (n=5-7) (significant versus MCF-7 cell- implanted group, ^**^P<0.01; compared to vehicle- treated, TAMR-MCF-7 cells- implanted group^##^P<0.01). **(H)** Representative pictures of tumor growth formed by CAMs bearing MCF-7 and TAMR-MCF-7 cells. **(I)** Tumor weight. Treatment with ketoconazole or 14,15-EEZE reduced tumor weight formed by CAMs bearing TAMR-MCF-7 cells. Data represent mean±SD (n=5-7) (significant versus MCF-7 cells-implanted group, ^**^P<0.01; compared to vehicle- treated, TAMR-MCF-7 cells- implanted group^##^P<0.01).

Vascular endothelial growth factor (VEGF) plays an important role in the angiogenic activity of diverse tissues [23, 24]. We previously showed that TAMR-MCF-7 cells exhibit increased VEGF-mediated angiogenesis compared to MCF-7 cells [25]. Additionally, the pro-angiogenic activity of EETs is mediated in part by VEGF [23, 24]. We hypothesized that CYP3A4 overexpression and CYP3A4-catalyzed 11,12-EET synthesis are linked to excessive VEGF production in TAMR-MCF-7 cells. As expected, the enhanced VEGF mRNA expression and transcriptional activity was diminished by ketoconazole and 14,15-EEZE in TAMR-MCF-7 cells (Figures 3A, 3B, and 3C). ELISA confirmed that treatment with ketoconazole and 14,15-EEZE significantly reduced the higher secretion of VEGF in TAMR-MCF-7 cells (Figure 3D and 3E).

We then tested the effect of these chemicals on the angiogenesis of TAMR-MCF-7 cells using a chick chorioallantoic membrane (CAM) assay, which is a convenient model used to evaluate tumor-induced angiogenesis [26]. CAM-bearing TAMR-MCF-7 cells had a higher intensity of blood vessels than CAM-bearing MCF-7 cells or VEGF (20 ng/CAM)-treated cells (Figure 3F and 3G). Treatment with ketoconazole or 14,15-EEZE significantly reduced the number of blood vessels around the tumor produced by TAMR-MCF-7 cells in a concentration-dependent manner (Figure 3F and 3G). Moreover, we also found that the tumors formed from TAMR-MCF-7 cell masses in CAM were much larger than those from MCF-7 cells, and that the tumor weights were significantly decreased by ketoconazole or 14,15-EEZE treatment (Figure 3H and 3I). These data highlight a critical role of CYP3A4 overexpression and EET formation in the regulation of both VEGF-mediated angiogenesis and tumorigenesis in TAM-resistant breast cancer.

### Effects of a CYP3A4 inhibitor and an EET antagonist on cell migration of TAMR-MCF-7 cells

**Figure 4:**
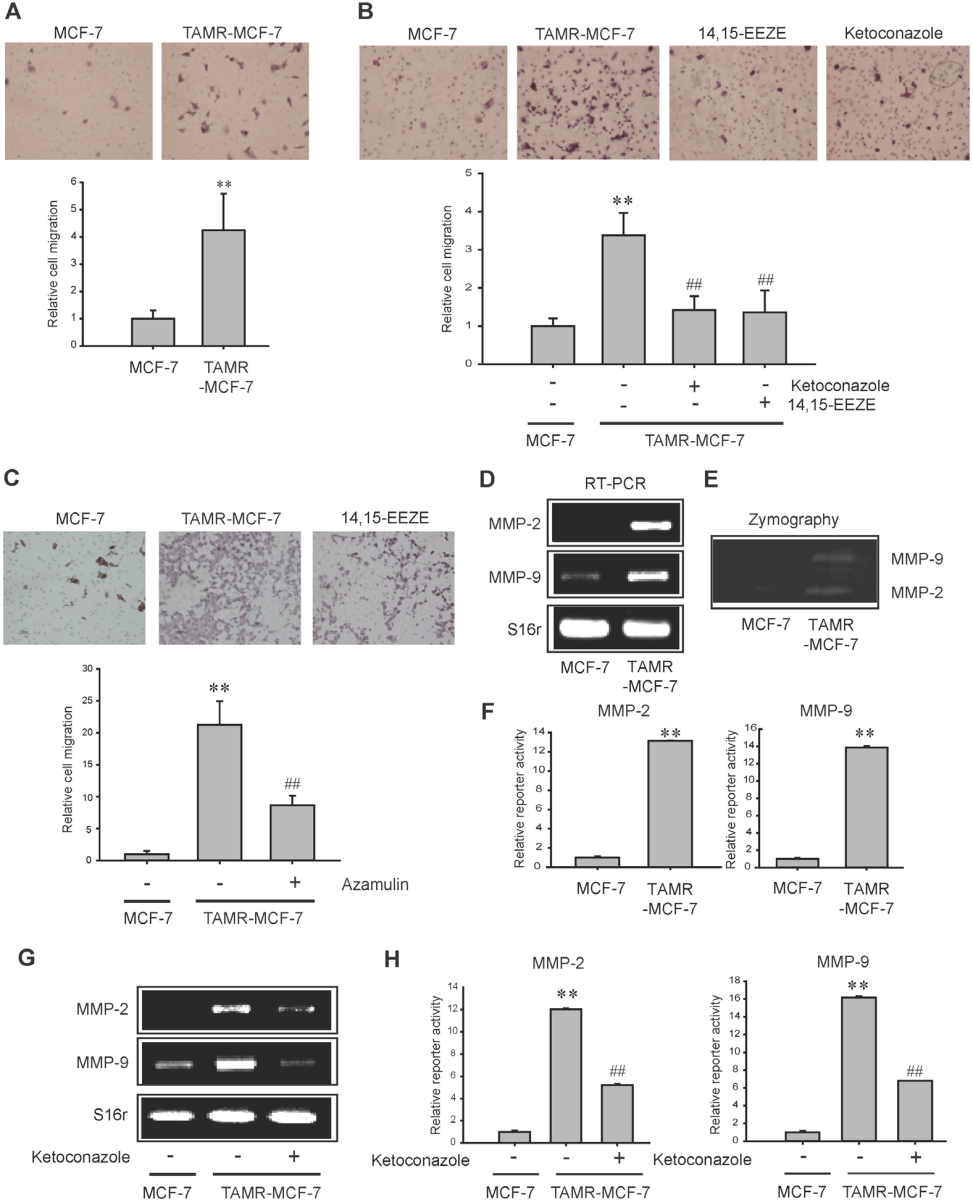
Effects of CYP3A4 inhibitor and EET antagonist on cell migration of TAMR-MCF-7 cells **(A)** Transwell migration assays were performed with MCF-7 cells and TAMR-MCF-7 cells for 17 h. Represestative figures of migrated cells (left). The relative cell numbers of migrated cells between MCF-7 cells and TAMR-MCF-7 cells (right). Values are mean±SD (n= 8-10) (significant versus MCF-7 cells, ^**^P<0.01). **(B)** Inhibitory effect of ketoconazole (10 μM) or 14,15-EEZE (3 μM) on cell migration of TAMR-MCF-7 cells. Represestative figures of migrated cells (left). The relative cell numbers of migrated cells (right). Values are mean ±SD (n= 8-10) (significant versus MCF-7 cells, ^**^P<0.01; significant versus vehicle treated TAMR-MCF-7 cells, ^##^P<0.01). **(C)** Effect of azamulin on cell migration of TAMR-MCF-7 cells. Values are mean±SD (n=8) (significant versus MCF-7 cells, ^**^P<0.01; significant versus vehicle treated TAMR-MCF-7 cells, ^##^P<0.01). **(D)** Basal levels of MMP-2 and MMP-9 mRNA in MCF-7 and TAMR-MCF-7 cells. **(E)** The activites of MMP-2 and MMP-9 were measured in serum-free medium by gelatin zymography assay in MCF-7 and TAMR-MCF-7 cells. **(F)** Reporter gene assays using MMP-2 and MMP-9 promoter reporters. Luciferase reporter gene assays were performed to detect promoter activities of MMP-2 and MMP-9. Each cell type was transiently co-transfected with MMP-2-luc reporter plasmid or MMP-9-luc reporter plasmid (1 μg/ml) and phRL-SV (hRenilla) (1 ng/ml). Dual luciferase reporter assays were performed as described in Figure 3B or 3C. Data represent mean±SD with 4 different samples (significant versus MCF-7 cells, ^**^P<0.01). **(G)** Effect of ketoconazole (10 μM) on mRNA levels of MMP-2 and MMP-9 in TAMR-MCF-7 cells. **(H)** Effect of ketoconazole (10 μM) on the basal reporter activity of MMP-2 and MMP-9 in TAMR-MCF-7 cells. Each cell type was transiently co-transfected with MMP-2-luc reporter plasmid or MMP-9-luc reporter plasmid (1 μg/ml) and phRL-SV (hRenilla) (1 ng/ml). Dual luciferase reporter assays were performed as described in Figure 3B or 3C. Data represent mean±SD with 3 different samples (significant versus MCF-7 cells, ^**^P<0.01; significant versus vehicle treated TAMR-MCF-7 cells, ^##^P<0.01).

CYP epoxygenase and EET production have been found to correlate with human cancer metastasis [17]. Specifically 11,12-EET contributes to the migration and invasion capabilities of prostate cancer [15]. Hiscox *et al.* reported that breast cancer cells exhibit greater metastatic ability, characterized by increased motile and invasion behavior during the acquisition of tamoxifen resistance [27, 28]. Here, we confirmed that TAMR-MCF-7 cells display enhanced migration ability compared to MCF-7 cells (Figure 4A). We then tried to identify a role for CYP3A4-mediated 11,12-EET production in the cellular migration of TAMR-MCF-7 cells. Treatment with ketoconazole, azamulin and 14,15-EEZE significantly inhibited the cellular migration of TAMR-MCF-7 cells (Figure 4B and 4C). These data suggest that up-regulated CYP3A4-dependent 11,12-EET formation is related to a greater migration capacity of TAMR-MCF-7 cells.

Cancer metastasis is associated with enhanced synthesis of metastasis-related genes, including matrix metalloproteinases (MMPs), a family of proteinases [29, 30]. High levels of MMP-2 and MMP-9 have been related to breast cancer invasion [30]. We further examined the expression of MMP-2 and MMP-9 in both cell lines. An increase in mRNA expression and the enzymatic activities of both MMP-2 and MMP-9 were observed in TAMR-MCF-7 cells compared to MCF-7 cells by RT-PCR (Figure 4D) and zymography analyses (Figure 4E). Moreover, reporter gene assays with human MMP-2 and MMP-9 promoter-luciferase constructs also revealed that the transcription of both genes was increased in TAMR-MCF-7 cells (Figures 4F). To explore whether CYP3A4-mediated EET production is involved in the up-regulation of MMP-2 and MMP-9 in TAMR-MCF-7 cells, cells were exposed to ketoconazole for 24 h. The mRNA levels (Figure 4G) and reporter gene activities (Figures 4H) of both MMP-2 and MMP-9 were suppressed by ketoconazole, suggesting that CYP3A4-dependent 11,12-EET production may be related to the up-regulation of MMPs and migratory phenotypic changes in TAMR-MCF-7 cells.

### Influence of CYP3A4-mediated EET production on Pin1 expression in TAMR-MCF-7 cells

**Figure 5:**
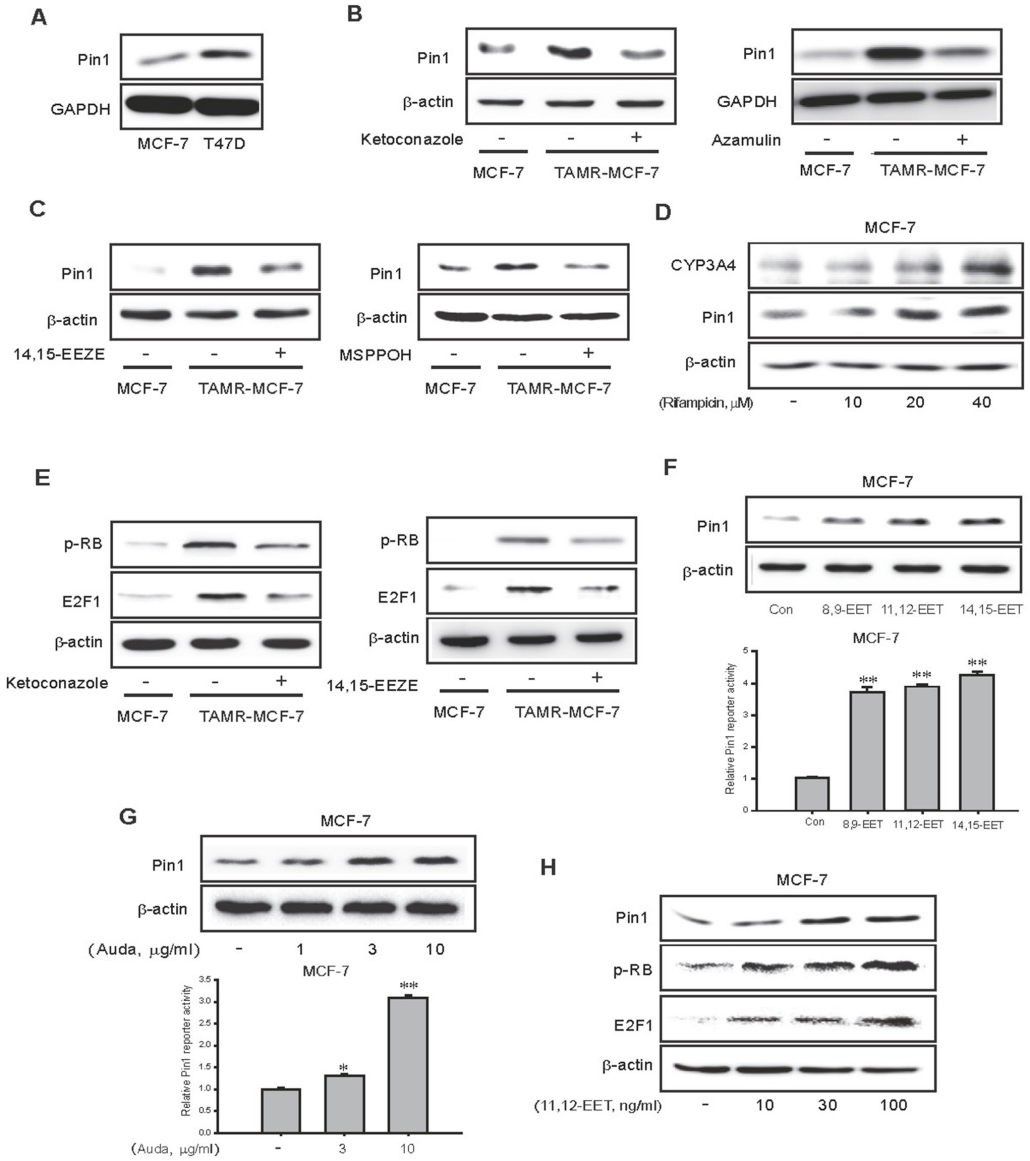
Effects of CYP3A4 inhibitor and EET antagonist on RB/E2F1-dependent Pin 1 expression in TAMR-MCF-7 cells **(A)** Comparison of Pin1 expression in MCF-7 and T47D cells **(B)** Effects of ketoconazole and azamulin on the expression of Pin1 protein in TAMR-MCF-7 cells. Pin1 overexpression in TAMR-MCF-7 cells was suppressed by treatment of ketoconazole (10 μM) and azamulin (10 μM) for 24 h. **(C)** Effects of EET antagonist and epoxygenase inhibitor on the expression of Pin1 protein in TAMR-MCF-7 cells. TAMR-MCF-7 cells were incubated with 14,15-EEZE (10 μM) and MSPPOH (10 μg/ml) for 24 h. **(D)** Effect of rifampicin on CYP3A4 and Pin1 expression in MCF-7 cells. MCF-7 cells were incubated with 10-40 μM rifampicin for 24 h. **(E)** Effect of ketoconazole (10 μM; left) or 14,15-EEZE (10 μM; right) on the elevated phosphorylation of RB and the enhanced expression of E2F1 protein in TAMR-MCF-7 cells. **(F)** Effect of EETs on expression of Pin 1 protein (upper) and basal activity of Pin1 promoter reporter (lower) in MCF-7 cells. MCF-7 cells were exposed with 8,9-EET, 11,12-EET and 14,15-EET (100 ng/ml, respectively) for 24 h. Protein levels and reporter activities of Pin 1 were determined by western blotting and reporter gene assay. **(G)** Effect of Auda, a sEH inhibitor on protein expression of Pin1 (upper) and the basal activity of Pin1 promoter (lower) in MCF-7 cells. MCF-7 cells were exposed to Auda for 24 h in the indicated concentration. Protein level and reporter activity of Pin 1 were determined by western blotting and reporter gene assay. **(H)** Effects of 11,12-EET treatment on the protein expression of Pin1, phosphorylated RB and E2F1 in MCF-7 cells. MCF-7 cells were treated with 11,12-EET for 24 h in the indicated concentration.

Pin1, a peptidyl prolyl isomerase, is an enzyme that is crucial for diverse cancer developmental processes including proliferation, apoptosis, and migration [31]. We previously reported that Pin1 overexpression is critical for VEGF-mediated angiogenesis and the EMT process in TAMR-MCF-7 cells [25, 32]. The basal expression of Pin1 was also higher in relatively TAM-resistant T47D cells than MCF-7 cells (Figure 5A). Given the important role of Pin1 in angiogenesis and cell migration, we further examined a possible relationship between Pin1 and CYP3A4-mediated 11,12-EET production in TAMR-MCF-7 cells. Treatment with ketoconazole, azamulin and 14,15-EEZE significantly reduced the protein expression of Pin1 in TAMR-MCF-7 cells (Figure 5B and left panel of 5C). Furthermore, treatment with MSPPOH, an epoxygenase inhibitor, also suppressed the level of Pin1 (right panel of Figure 5C). Vice versa, Incubation of MCF-7 cells with 40 μM rifampicin, a potent CYP3A4 inducer for 24 h, the protein levels of CYP3A4 and Pin1 were enhanced compared to vehicle-treated control (Figure 5D). Rb1-dependent E2F1 activity is required for Pin1 gene transcription in TAMR-MCF-7 cells [33]. We also found that exposure of TAMR-MCF-7 cells to ketoconazole or 14,15-EEZE inhibited Rb phosphorylation and reduced the protein expression of E2F1 (Figure 5E). These data suggest that the diverse effects of CYP3A4-mediated 11,12-EET production in TAMR-MCF-7 cells may be linked to Rb/E2F1-dependent Pin1 activity.

To confirm these findings, MCF-7 cells were exposed to various EETs, including 8,9-EET, 11,12-EET, and 14,15-EET (100 ng/ml, respectively). All EETs significantly increased Pin1 expression and Pin1 gene promoter activity in MCF-7 cells (Figure 5F). Additionally, AUDA, a selective soluble epoxide hydrolase inhibitor that inhibits the biological metabolism of EETs, also caused a marked increase in Pin1 expression and Pin1 reporter activity in MCF-7 cells (Figure 5G). Moreover, exposure to 11,12-EET, which has been identified as a major EET product in TAMR-MCF-7 cells, increased Pin1 and Rb/E2F1 expression in a concentration-dependent manner in MCF-7 cells (Figure 5H).

**Figure 6:**
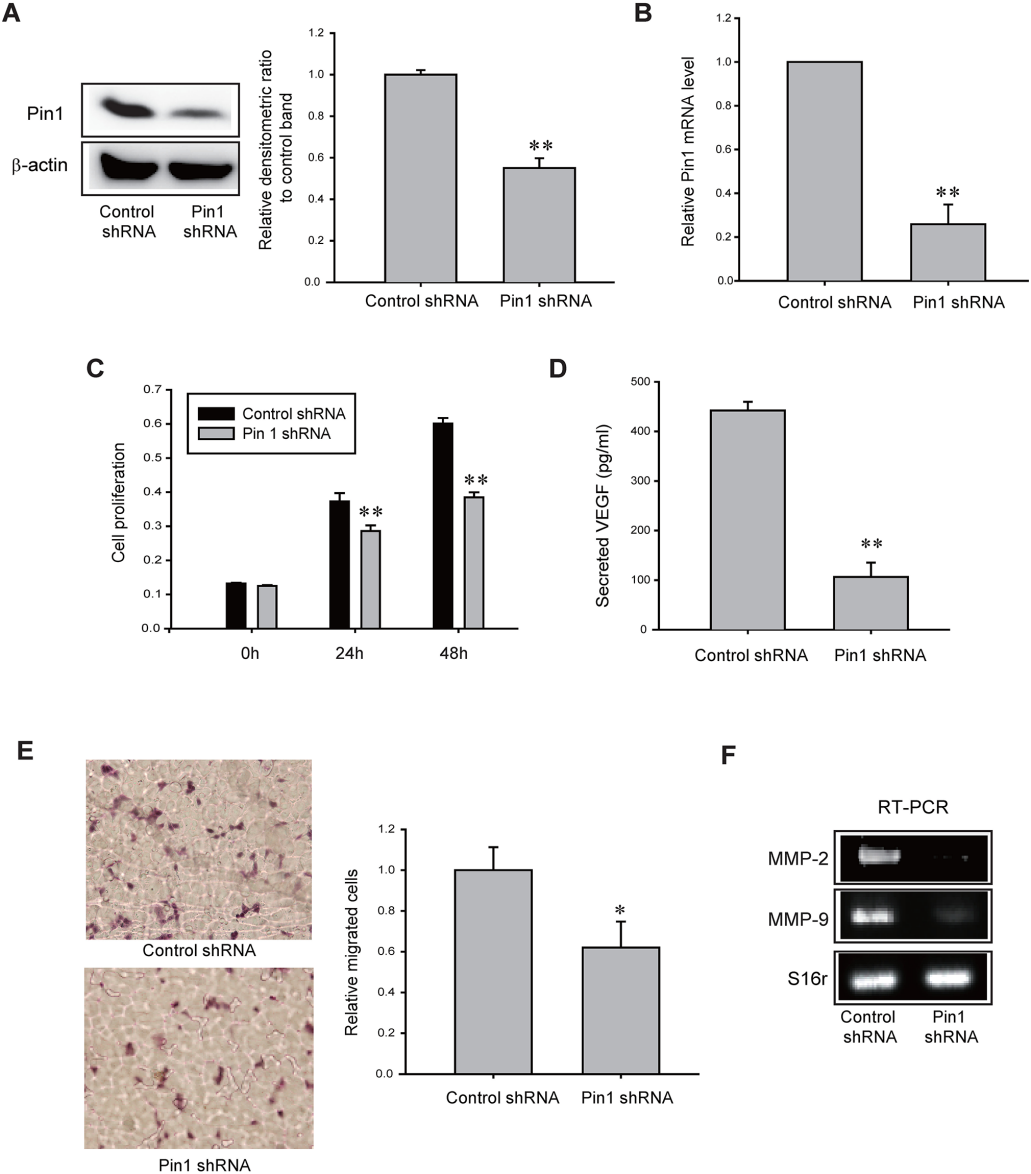
Role of Pin1 in cell proliferation, VEGF production and cell migration of TAMR-MCF-7 cells **(A, B)** Protein (A) and mRNA (B) levels of Pin1 in control shRNA- and Pin1 shRNA-infected TAMR-MCF-7 cells. Pin 1 mRNA levels were measured by quantitative real-time PCR. **(C)** Comparison of cell proliferation between Pin1 shRNA/TAMR-MCF-7 cells (Pin1-knockdown) and control shRNA/TAMR-MCF-7 cells. Values are mean±SD with 8 different samples (significant versus control shRNA/TAMR-MCF-7 cells, ^**^P<0.01). **(D)** VEGF secretion in control shRNA/TAMR-MCF-7 cells and Pin1 shRNA/TAMR-MCF-7 cells. Values are mean±SD with 3 different samples (significant versus control shRNA/TAMR-MCF-7 cells, ^**^P<0.01). **(E)** Migration of control shRNA/TAMR-MCF-7 cells and Pin 1 shRNA/TAMR-MCF-7 cells. Transwell migration assays were performed with both cell lines for 17 h. Representative figures of migrated cells (left). The relative cell numbers of migrated cells between control shRNA/TAMR-MCF-7 cells and Pin1 shRNA/TAMR-MCF-7 cells (right). Values are mean±SD (n=8-10) (significant versus control shRNA/TAMR-MCF-7 cells, ^*^P<0.05). **(F)** mRNA levels of MMP-2 and MMP-9 in control shRNA/TAMR-MCF-7 cells and Pin1 shRNA/TAMR-MCF-7 cells.

Finally, to assess whether CYP3A4/EET-mediated Pin1 activation is a cause of increased proliferation, migration, and VEGF secretion in TAMR-MCF-7 cells, we established TAMR-MCF-7 cells stably expressing Pin1 shRNA. The infection efficacy was confirmed by a decrease in the protein and mRNA expression levels of Pin1 (Figure 6A and 6B). Similar to the effects of the CYP3A4 inhibitor and the EET antagonist, Pin1 knock-down TAMR-MCF-7 cells had a slower proliferation rate than control shRNA/TAMR-MCF-7 cells (Figure 6C). Moreover, VEGF secretion in TAMR-MCF-7 cells was diminished by Pin1 knock-down (Figure 6D). Pin1 silencing also inhibited cell migration of TAMR-MCF-7 cells (Figure 6E) and reduced the mRNA levels of MMP-2 and MMP-9 (Figure 6F).

## DISCUSSION

The pathological consequences of cancer are mainly correlated to tumor growth and metastasis, which are caused by uncontrolled cell proliferation, invasion, and migration. Angiogenesis is also implicated in the growth of neoplastic tumors, although it is critical for the processes of wound healing and tissue regeneration [8]. TAM-resistant breast cancer exhibits typical characteristics of aggressive cancer, including rapid growth, microvessel formation, and a high capacity for metastasis [27, 32, 34]. However, the underlying mechanisms for the malignant changes are poorly understood. Increasing evidence demonstrates that EET production, which is catalyzed by CYP epoxygenases, is involved in the proliferation and migration of cancer cells [8, 35, 36]. A recent paper also reported that EET levels are enhanced in human breast cancer tissues, which is associated with aggressive behavior [37]. Here, we found for the first time that overexpression of CYP3A4 epoxygenase is involved in cell proliferation, angiogenesis, and migration in TAM-resistant human breast cancer cells through the biosynthesis of 11,12-EET.

CYP3A4 is mainly expressed in human liver tissue and is considered the most important enzyme in the biotransformation of xenobiotics including various anticancer drugs [38]. CYP3A4 is also expressed in several cancer cell lines such as breast, colon and liver [36, 39, 40]. It has been reported that several anti-cancer agents such as tamoxifen can induce CYP3A4 in cancer cells, which is related to the acquisition of chemoresistance [41, 42]. In our study, CYP3A4 was overexpressed in TAM-resistant breast cancer cells, and its enzymatic inhibition significantly reduced cell proliferation and induced synergistic apoptosis with 4-hydroxytamoxifen. Hence, the up-regulation of CYP3A4 in TAMR-MCF-7 cells may be important for both exaggerated cell growth and the impaired response to chemotherapy. Moreover, the enhanced angiogenesis and migration observed in TAMR-MCF-7 cells were dramatically suppressed by CYP3A4 inhibition, suggesting a critical role of CYP3A4 in malignant changes during the development of TAM resistance. A previous study reported that EET produced from CYP2J2 is important for cancer metastasis in breast cancer [17]; however, other studies have reported that CYP2J2 is not associated with overall survival, and that its immunoactivity is nearly undetectable in breast cancer tissues [18, 19]. Compared to the parental MCF-7 cells, CYP2J2 protein was not detected and its mRNA level was even decreased in TAMR-MCF-7 cells, suggesting that CYP2J2 is unrelated to malignant changes in resistant cells. A recent study using human breast cancer and adjacent noncancerous tissues revealed that CYP2C8 and 2C9 protein levels positively correlated with the intensity of Ki67, a representative cellular proliferation marker protein, and that CYP2J2 levels positively correlated with histological grade and tumor size [37]. In the present study, we found that CYP3A4 was highly expressed in TAMR-MCF-7 cells, while the protein and mRNA levels of CYP2C8 and 2C9 were limited. Hence, these results highlight a pivotal role of CYP3A4 up-regulation in multiple regulation of cancer aggressiveness in TAM-resistant breast cancer.

EETs play an important role in cancers due to their ability to promote cell proliferation, angiogenesis and metastasis [17, 35]. Here, we demonstrated a selective increase in 11,12-EET biosynthesis in TAMR-MCF-7 cells. Additionally, blocking the function of EETs by an EET antagonist yielded results similar to those obtained following the inhibition of CYP3A4 activity with respect to cell proliferation, angiogenesis, and migration of TAMR-MCF-7 cells. These data suggest that the effects of CYP3A4 in TAMR-MCF-7 cells are in part related to 11,12-EET production. Studies using purified CYP epoxygenases have indicated that the catalytic activity and regio-selectivity of EET formation are P450 isoform- and species-dependent [7, 43, 44]. Each enzyme is able to convert AA to all four EET regioisomers, and the main products in many cases are 11,12-EET and 14,15-EET [6]. In the case of CYP2C8 or CYP2C9, 14,15-EET production is dominant compared to 11,12-EET or 8,9-EET [7, 45, 46]. 11,12-EET is known to be a major product of CYPs in prostate cancer [15]. Our results support explicating the selective 11,12-EET biosynthesis through the up-regulation of CYP3A4 in TAM-resistant breast cancer. However, the mechanisms for the selective biosynthesis and metabolism of eicosanoids in TAMR-MCF-7 cells require further study.

EETs promote angiogenesis via mitogen-activating protein kinase-dependent eNOS up-regulation [47] or Src/STAT3-dependent VEGF expression [48]. It has also been shown that 11,12-EET stimulates angiogenesis by activating epidermal growth factor receptor (EGFR) [49] and sphingosine kinase 1 [50]. Most of these studies have reported that the pro-angiogenic activity of EETs is mediated through VEGF production [23, 24]. Webler *et al.* suggested a positive feedback loop between VEGF and EET production in endothelial cells [23]. VEGF induces CYP2C8 gene expression via transcriptional activation, which causes increased intracellular EET levels [23]. We previously reported that VEGF overexpression is required for enhanced angiogenesis in TAMR-MCF-7 cells [25]. Here, we demonstrate that CYP3A4-catalyzed 11,12-EET stimulates angiogenic progression via VEGF up-regulation in TAMR-MCF-7 cells. Hence, EET signaling seems to be involved in the enhanced angiogenesis in TAM-resistant breast cancer.

CYP epoxygenases and their derived EETs stimulate the migration and invasion of various cell types through the up-regulation of the pro-metastatic MMPs and CD44, and down-regulation of the anti-metastatic genes CD82 and NM23 [17]. Additionally, 11,12-EET increases cell motility via EGFR and subsequent Akt activation in prostate carcinoma cancer [15]. The findings of the present study indicate that CYP3A4-dependent 11,12-EET formation contributes to enhanced migration capacity of TAMR-MCF-7 cells and that the effect may be related to the activities of MMP-2 and MMP-9, because CYP3A4 inhibition abrogates the expression of both metalloproteinases. Several reports have already shown that EETs directly enhance the expression of MMP-2 and MMP-9 in neutrophil and wound tissues [51, 52]. Moreover, EET antagonist attenuates wound closure in sEH-null mice [52].

Overexpression of Pin1, a peptidyl-prolyl isomerase protein, is closely related to the exaggerated cell proliferation and acquired drug resistance in TAMR-MCF-7 cells [53, 54]. Moreover, Pin1 functions as an essential protein required for angiogenesis [25], EMT-like behavior [32] and migration/invasion of cancer cells [55], all of which may contribute to the metastatic ability of TAM-resistant breast cancer. Because the biological function of Pin1 appears to strongly resemble that of EET in TAMR-MCF-7 cells, we hypothesized that 11,12-EET up-regulates Pin1 status according to cell type. With regard to cell proliferation rate, VEGF production, and the migration ability of TAMR-MCF-7 cells, the results from Pin1 knock-down experiments were similar to those from the inhibitor studies on CYP3A4 and EET. Moreover, the CYP3A4-mediated 11,12-EET pathway participated in Rb/E2F1-dependent Pin1 gene transcription. Our data suggest that Pin1 acts as a key mediator for the effects of CYP3A4-derived 11,12-EET biosynthesis in TAM-resistant breast cancer.

Overall, our findings demonstrated for the first time that selective overexpression of CYP3A4 and 11,12-EET production in TAMR-MCF-7 cells play pivotal roles in regulating malignant changes including rapid growth, angiogenesis, and migration. Our findings suggest that CYP3A4 inhibitors and EET antagonists may serve as novel therapeutic agents for the treatment of TAM-resistant breast cancer.

## MATERIALS AND METHODS

### Materials

Antibodies against CYP3A4, CYP2C8, and CYP2C9 were purchased from Abcam (Cambridge, UK). Antibodie against p-RB were obtained from Cell Signaling Technology (Beverly, MA, USA). Antibodies against Pin 1 and E2F1 were purchased from Santa Cruz Biotechnology (Santa Cruz, CA). Horseradish peroxidase-conjugated donkey anti-rabbit, anti-goat IgG, and alkaline phosphatase-conjugated donkey anti-mouse IgG were acquired from Jackson Immunoresearch Laboratories (West Grove, PA). Anti-actin antibody and most of the reagents used for molecular studies were obtained from Sigma (St. Louis, MO). VEGF-luc plasmid was kindly donated from Dr. Lee (Chonnam National University, Gwangju, Korea). Full length human MMP-2 and MMP-9 promoter luciferase constructs were kindly provided by Dr. Aree Moon (Duksung women’s University, Seoul, Korea). Ketoconazole was obtained from Sigma (St. Louis, MO) and N-(methylsulfonyl)-2-(2-propynyloxy)-benzenehexanamide (MSPPOH), 14,15-EEZE, 8,9-EET, 11,12-EET, 14,15-EET and 12-(3-Adamantan-1-yl-ureido)-dodecanoic acid (AUDA) were purchased from Cayman Chemical Co. (Ann Arbor, MI). 6*β*-hydroxytestosterone was obtained from BD Gentest Co. (Woburn, MA).

### Cell culture and establishment of tamoxifen-resistant MCF-7 (TAMR-MCF-7) cells

ER-positive MCF-7 cells were cultured at 37°C in 5% CO_2_/95% air in Dulbecco’s modified Eagle’s medium (DMEM) containing 10% fetal bovine serum (FBS), 100 units/ml penicillin, and 100 μg/ml streptomycin. TAMR-MCF-7 cells were established using methods previously reported [34, 56] and cultured in DMEM containing 10% charcoal-stripped FBS (Hyclone, Logan, UT) and 4-hydroxytamoxifen (3 μM). For the experiment purpose, the chemicals were exposed in serum free medium condition. For generation of stable knockdown TAMR-MCF-7 cell lines, the lentiviral transduction particles containing short hairpin RNA (shRNA) for Pin 1 (sc-36230-v), or nontarget control shRNA (sc-108080) were purchased from Santa Cruz. Cells were transduced with virus in the presence of polybrene (5 μg/mL) for 24 h and then selected by puromycin (3 μg/mL).

### Immunoblot analysis

After washing with sterile PBS, cells were lysed in lysis buffer containing 20 mM Tris-Cl (pH 7.5), 1% Triton X-100, 137 mM sodium chloride, 10% glycerol, 2 mM EDTA, 1 mM sodium orthovanadate, 25 mM β-glycerolphosphate, 2 mM sodium inorganic pyrophosphate, 1 mM phenylmethylsulfonylfluoride, and 1 μg/ml leupeptin. Total cell lysates were centrifuged at 10,000*g* for 10 min to remove cell debris, and proteins in the supernatant were fractionated using a 12% separating gel. The fractionated proteins were then transferred electrophoretically to nitrocellulose paper, and the proteins were immunoblotted with specific antibodies.

### CYP3A4 activity assay

To determine CYP3A4 activity, MCF-7 and TAMR-MCF-7 cells were incubated with 200 μM testosterone for 2, 4 or 6 h at 37°C. 100 μL aliquot was collected in a 1.5-mL microcentrifuge tube containing an identical volume of ice-cold acetonitrile–methanol (1:1) containing carbamazepine (100 nM) as an internal standard and cells were harvested using non-denaturing lysis buffer for the determination of cellular protein. CYP3A4-dependent 6*β*-hydroxytestosterone formation was determined as described in a previous paper [57].

### Reporter gene analysis

A dual-luciferase reporter gene assay system (Promega, Madison, WI) was used to determine promoter activity. Briefly, cells were plated in 12-well plates and transiently transfected with 1 μg/ml reporter plasmids and phRL-SV plasmid (hRenilla luciferase expression for normalization) using Hillymax® reagent (Dojindo Molecular Technologies, Gaithersburg, MD). The cells were then incubated in culture medium without serum for 18 h. Firefly and hRenilla luciferase activities in the cell lysates were measured using a luminometer (LB941, Berthold Technologies, Bad Wild, Germany). Relative luciferase activities were calculated by normalizing the promoter-driven firefly luciferase activity to the hRenilla luciferase.

### EET extraction from breast cancer cells and determination by LC-ESI/MRM/MS Method

Cells were collected in cold PBS containing 2 μM soluble epoxide hydrolase inhibitor 1471 (a gift from Dr. Bruce Hammock, University of California, Davis, CA). The pellets were extracted with a 1:1 mixture of methanol/chloroform (1.32 ml) for 1 min, and 0.33 ml of distilled water was added and the mixture was vortexed for additional 1 min. After 60 min, the samples were then separated into two layers by centrifugation for 20 minutes at 15,800 g at 4 °C. The lower layer was evaporated with vacuum centrifugation, and the residues were dissolved in 20 μl methanol for mass spectrometric analysis. Two microlitter of samples were subjected to LC-ESI/MRM/MS analysis with API 2000 Mass Spectrometer (AB/SCIEX) coupled with an Agilent 1100 HPLC (Agilent, CO) using nitrogen as the collision gas (heater turbo gas temperature: 500 °C). Negative ion monitoring was performed with the following diagnostic product ions: 319 *m*/*z*3 127*m*/*z* for 8,9-EET; 319 *m*/*z*3 167 *m*/*z* for 11,12-EET; 319 *m*/*z*3 219 *m*/*z* for 14,15-EET. Base-line resolution of EET regioisomers was achieved on a Kinetex C18 analytical column (100 mm × 4.6 mm, 2.6 μm; Phenomenex, Torrance, CA) using the following mobile phase combinations-linear gradient: 10% B (0 min), 90% B (8 min), 95% B (8.1 min), 95% B (18 min), 10% B (20 min), an addition of 10% B (30 min); A: Distilled water, B: Acetonitrile: Methanol (88:12, v/v); 800 μl/min flow rate.

### Cell proliferation

After exposure of cells to 10% FBS containing medium for the indicated time, viable adherent cells were stained with MTT [3-(4,5-dimethylthiazol-2-yl)-2,5-diphenyl-tetrazolium bromide](2 mg/mL) for 4 h. Media were then removed and the formazan crystal-stained cells were dissolved in 200 μl dimethylsulfoxide. Absorbance was assayed at 540 nm using a microtiter plate reader (Berthold Tech., Bad Wildbad, Germany).

### Flow cytometry

TAMR-MCF-7 cells were incubated in serum-free DMEM with 3 or 10 μM 4-hydroxytamoxifen in the presence or absence of ketoconazole (10 μM) or 14,15-EEZE (3 μM) for 24 h. The cells were harvested with trypsin treatment, stained with both annexin V–FITC and propidium iodide according to the manufacture’s protocol (Invitrogen, Carlsbad, CA) and analysed by flow cytometry (FACStar, BD Biosciences, Mississauga, ON) set for FL1 (annexin V) and FL2 (propidium iodide). A total of 10^4^ cells were counted for each sample.

### Vascular endothelial growth factor (VEGF) enzyme-linked immunosorbent assay (ELISA)

A commercial ELISA kit (Biosource Diagnostics, Nivelles, Belgium) was used to determine VEGF concentrations in media according to the manufacturer's protocol. Briefly, cells were plated in six-well culture plates and incubated in serum-free medium for 24 h, and then the culture medium was measured with ELISA. VEGF concentrations were determined by measuring the absorbance at 420 nm and were normalized to total protein concentrations in each well.

### Migration assay

An *in vitro* migration assay was performed using a 24-well Transwell unit with polycarbonate filters (Corning Costar, Cambridge, MA) as previously described [58, 59]. The lower side of the filter was coated with type I collagen (Collaborative Research, Lexington, KY). The lower compartment was filled with 10% FBS. Ketoconazole, azamulin and 14,15-EEZE were added to the Transwell insert in the lower well. Cells were placed in the upper part of the Transwell plate, incubated for 17 h, fixed with formalin and methanol and stained with Hematoxylin for 10 min followed by a brief staining with Eosin. The migration phenotypes were determined by counting the cells that migrated to the lower side of the filter, using microscopy at 40× magnification. Eight fields were counted for each filter.

### Reverse transcriptase-polymerase chain reaction (RT-PCR)

Total RNA was isolated from the cells using a total RNA isolation kit (RNAgents, Promega, Madison, WI). The total RNA (1.0 μg) was reverse transcribed using an oligo(dT) 18-mer and Moloney murine leukemia virus reverse transcriptase (Bioneer). PCR was done using selective primers for human CYP3A4 (Forward, CATTCCTCATCCCAATTCTTGAAGT; Reverse, CCACTCGGTGCTTTTGTGTATCT), CYP2C8 (Forward, AGATCAGAATTTTCTCACCC; Reverse, AACTTCGTGTAAGAGCAACA), CYP2C9 (Forward, AGGAAAAGCACAACCAACCA; Reverse, TCTCAGGGTTGTGCTTGTC), VEGF (sense primer, 5′-GCTACTGCCATCCAATCGAG-3′; antisense primer, 5′-TGCATTCACATTTGTTGTGC-3′), MMP-2 (Forward, AGTCTGAAGAGCGTGAAG; Reverse, CCAGGTAGGAGTGAGAATG), MMP-9 (Forward, TGACAGCGACAAGAAGTG; Reverse, CAGTGAAGCGGTACATAGG) and S16 ribosomal protein (S16r) genes (sense, 5′-TCCAAGGGTCCGCTGCAGTC-3′; antisense, 5′-CGTTCACCTTGATGAGCCCATT-3′). PCR was carried out for 40 cycles under the following conditions: denaturation at 95°C for 10 s, annealing at 52°C for 30 s, and elongation at 72°C for 1 min. The band intensities of the amplified DNA were compared after visualization with an FLA-7000 (Fujifilm, Tokyo, Japan).

### Realtime quantitative PCR (qPCR)

Total RNA was isolated by using Trizol (Invitrogen, USA). qPCR was performed with a real time PCR machine and primers specific for Pin1 (Forward, TCGGGAGAGGAGGACTTTG; Reverse, GGAGGATGATGTGGATGCC) and GAPDH (Forward, CATGAGAAGTATGACAACAGCC; Reverse, AGTCCTTCCACGATACCAAAG).

### Gelatin zymography

Enzymatic activity of MMP-2 and MMP-9 was measured by gelatin zymography in 10% SDS-polyacrylamide separating gels in the presence of 0.1% gelatin. Serum free media were collected and incubated with an adequate volume of SDS sample loading buffer for 10 minutes at room temperature, and separated at 4°C. Following electrophoresis, the gel was rinsed in enzyme renaturing buffer containing Triton X-100 (30 minutes at room temperature). Subsequently, the gel was incubated in developing buffer (50 mM TrisHCl, pH=7.5; 200 mM NaCl; 5 mM CaCl_2_; 0.02% Brij-35) overnight at 37°C, stained with Coomassie brilliant blue R-250 solution (0.5%) for 30 minutes, and then destained in methanol, acetic acid and water (5:4:1) solution until clear bands of MMP activity were visible on the dark blue background. Digestive activity of MMPs was confirmed by presence of two bands on zymograms, slower migrating MMP-9 (94 kDa) and faster migrating MMP-2 (72 kDa).

### Chick chorioallantoic membrane assay

Chick chorioallantoic membrane (CAM) assays were done according to previously published methods [60, 61]. The surfaces of 10 days-old post-fertilization chick eggs were sterilized and the CAM exposed by cutting a window (1 cm^2^) on one side of the egg using the false air sac technique. Both the MCF-7 and TAMR-MCF-7 cells (2x 10^6^ cells) were placed on the exposed CAM and the windows were sealed with transparent tape. VEGF (20 ng/mL) was used as a standard proangiogenic agent. The eggs were then incubated in a humidified incubator at 37°C and treated with ketoconazole and 14,15-EEZE with indicated doses. Eggs were examined every 72 h after inoculation using an SV6 stereomicroscope (Carl Zeiss, Oberkochen, Germany) at ×50 magnification. Digital images of CAM sections were collected using a three-charge-coupled device color video camera system. Images were analyzed using Image-Pro software (Media Cybernetics, Rockville, MD). The number of vessel branch points contained in a circular region was counted. The tumors were excised and measured that the size to evaluate the influence of chemicals on the tumorigenesis development.

### Statistical analysis

Scanning densitometry was done using an LAS-3000mini (Fujifilm, Tokyo, Japan). A paired Student’s t test was used to examine between group differences. Statistical significance was accepted at either *P* < 0.05 or *P* <0.01.

## Abbreviations

EETs: Epoxyeicosatrienoi acids
CYP: Cytochrome P450
TAM: Tamoxifen
4-OH-TAM: 4-hydroxy tamoxifent
15-EEZE: 14, 15-epoxyeicosa-5(Z)-enoic acid
MSPPOH: N-methylsulfonyl-6-(2-propargylozyphenyl) hexanamide
VEGF: Vascular endothelial growth factor
EMT: epithelial mesenchymal transitions
CAM assay: chick chorioallantoic membrane assay

## References

[1] Petrangeli E, Lubrano C, Ortolani F, Ravenna L, Vacca A, Sciacchitano S, Frati L, Gulino A (1994). Estrogen receptors: new perspectives in breast cancer management. The Journal of steroid biochemistry and molecular biology.

[2] Ali S, Coombes RC (2002). Endocrine-responsive breast cancer and strategies for combating resistance. Nature Reviews Cancer.

[3] Osborne CK, Fuqua SA (1994). Mechanisms of tamoxifen resistance. Breast cancer research and treatment.

[4] Guengerich FP, Shimada T (1991). Oxidation of toxic and carcinogenic chemicals by human cytochrome P-450 enzymes. Chemical research in toxicology.

[5] Gonzalez FJ, Gelboin HV (1994). Role of human cytochromes P450 in the metabolic activation of chemical carcinogens and toxins. Drug metabolism reviews.

[6] Capdevila JH, Falck JR, Harris RC (2000). Cytochrome P450 and arachidonic acid bioactivation: molecular and functional properties of the arachidonate monooxygenase. Journal of lipid research.

[7] Zeldin DC (2001). Epoxygenase pathways of arachidonic acid metabolism. Journal of Biological Chemistry.

[8] Xu X, Zhang XA, Wang DW (2011). The roles of CYP450 epoxygenases and metabolites, epoxyeicosatrienoic acids, in cardiovascular and malignant diseases. Advanced drug delivery reviews.

[9] Munzenmaier DH, Harder DR (2000). Cerebral microvascular endothelial cell tube formation: role of astrocytic epoxyeicosatrienoic acid release. American Journal of Physiology-Heart and Circulatory Physiology.

[10] Zhang C, Harder DR (2002). Cerebral capillary endothelial cell mitogenesis and morphogenesis induced by astrocytic epoxyeicosatrienoic acid. Stroke.

[11] Medhora M, Daniels J, Mundey K, Fisslthaler B, Busse R, Jacobs ER, Harder DR (2003). Epoxygenase-driven angiogenesis in human lung microvascular endothelial cells. American Journal of Physiology-Heart and Circulatory Physiology.

[12] Rifkind AB, Lee C, Chang TK, Waxman DJ (1995). Arachidonic acid metabolism by human cytochrome P450s 2C8, 2C9, 2E1, and 1A2:regioselective oxygenation and evidence for a role for CYP2C enzymes in arachidonic acid epoxygenation in human liver microsomes. Archives of biochemistry and biophysics.

[13] Lundblad MS, Stark K, Eliasson E, Oliw E, Rane A (2005). Biosynthesis of epoxyeicosatrienoic acids varies between polymorphic CYP2C enzymes. Biochemical and biophysical research communications.

[14] Ayajiki K, Fujioka H, Toda N, Okada S, Minamiyama Y, Imaoka S, Funae Y, Watanabe S, Nakamura A, Okamura T (2003). Mediation of arachidonic acid metabolite(s) produced by endothelial cytochrome P-450 3A4 in monkey arterial relaxation. Hypertens Res.

[15] Nithipatikom K, Brody DM, Tang AT, Manthati VL, Falck JR, Williams CL, Campbell WB (2010). Inhibition of carcinoma cell motility by epoxyeicosatrienoic acid (EET) antagonists. Cancer science.

[16] Oguro A, Sakamoto K, Funae Y, Imaoka S (2011). Overexpression of CYP3A4, but not of CYP2D6, Promotes Hypoxic Response and Cell Growth of Hep3B Cells. Drug Metabolism and Pharmacokinetics.

[17] Jiang JG, Ning YG, Chen C, Ma D, Liu ZJ, Yang S, Zhou J, Xiao X, Zhang XA, Edin ML (2007). Cytochrome p450 epoxygenase promotes human cancer metastasis. Cancer research.

[18] Mitra R, Guo Z, Milani M, Mesaros C, Rodriguez M, Nguyen J, Luo X, Clarke D, Lamba J, Schuetz E (2011). CYP3A4 mediates growth of estrogen receptor-positive breast cancer cells in part by inducing nuclear translocation of phospho-Stat3 through biosynthesis of (±)-14, 15-epoxyeicosatrienoic acid (EET). Journal of Biological Chemistry.

[19] Murray GI, Patimalla S, Stewart KN, Miller ID, Heys SD (2010). Profiling the expression of cytochrome P450 in breast cancer. Histopathology.

[20] Horwitz KB, Mockus MB, Lessey BA (1982). Variant T47D human breast cancer cells with high progesterone-receptor levels despite estrogen and antiestrogen resistance. Cell.

[21] Karey KP, Sirbasku DA (1988). Differential responsiveness of human breast cancer cell lines MCF-7 and T47D to growth factors and 17 beta-estradiol. Cancer Res.

[22] Murray GI, Patimalla S, Stewart KN, Miller ID, Heys SD (2010). Profiling the expression of cytochrome P450 in breast cancer. Histopathology.

[23] Webler AC, Michaelis UR, Popp R, Barbosa-Sicard E, Murugan A, Falck JR, Fisslthaler B, Fleming I (2008). Epoxyeicosatrienoic acids are part of the VEGF-activated signaling cascade leading to angiogenesis. American Journal of Physiology-Cell Physiology.

[24] Yang S, Wei S, Pozzi A, Capdevila JH (2009). The arachidonic acid epoxygenase is a component of the signaling mechanisms responsible for VEGF-stimulated angiogenesis. Archives of biochemistry and biophysics.

[25] Kim MR, Choi HS, Yang JW, Park BC, Kim JA, Kang KW (2009). Enhancement of vascular endothelial growth factor–mediated angiogenesis in tamoxifen-resistant breast cancer cells: role of Pin1 overexpression. Molecular cancer therapeutics.

[26] Ribatti D, Vacca A (1999). Models for studying angiogenesis *in vivo*. The International journal of biological markers.

[27] Hiscox S, Morgan L, Barrow D, Dutkowski C, Wakeling A, Nicholson RI (2004). Tamoxifen resistance in breast cancer cells is accompanied by an enhanced motile and invasive phenotype: Inhibition by gefitinib (Iressa', ZD1839). Clinical & experimental metastasis.

[28] Hiscox S, Jiang WG, Obermeier K, Taylor K, Morgan L, Burmi R, Barrow D, Nicholson RI (2006). Tamoxifen resistance in MCF7 cells promotes EMT-like behaviour and involves modulation of β-catenin phosphorylation. International Journal of Cancer.

[29] Stetler-Stevenson WG (1999). Matrix metalloproteinases in angiogenesis: a moving target for therapeutic intervention. Journal of Clinical Investigation.

[30] Sun Y, Lu N, Ling Y, Gao Y, Chen Y, Wang L, Hu R, Qi Q, Liu W, Yang Y (2009). Oroxylin A suppresses invasion through down-regulating the expression of matrix metalloproteinase-2/9 in MDA-MB-435 human breast cancer cells. European journal of pharmacology.

[31] Bao L, Kimzey A, Sauter G, Sowadski JM, Lu KP, Wang DG (2004). Prevalent overexpression of prolyl isomerase Pin1 in human cancers. Am J Pathol.

[32] Kim MR, Choi HK, Cho KB, Kim HS, Kang KW (2009). Involvement of Pin1 induction in epithelial–mesenchymal transition of tamoxifen-resistant breast cancer cells. Cancer science.

[33] Lee KY, Lee JW, Nam HJ, Shim JH, Song Y, Kang KW (2011). PI3-Kinase/p38 kinase-dependent E2F1 activation is critical for pin1 induction in tamoxifen-resistant breast cancer cells. Molecules and cells.

[34] Phuong NTT, Kim SK, Lim SC, Kim HS, Kim TH, Lee KY, Ahn SG, Yoon JH, Kang KW (2011). Role of PTEN promoter methylation in tamoxifen-resistant breast cancer cells. Breast cancer research and treatment.

[35] Jiang JG, Chen CL, Card JW, Yang S, Chen JX, Fu XN, Ning YG, Xiao X, Zeldin DC, Wang DW (2005). Cytochrome P450 2J2 promotes the neoplastic phenotype of carcinoma cells and is up-regulated in human tumors. Cancer research.

[36] Chen C, Wei X, Rao X, Wu J, Yang S, Chen F, Ma D, Zhou J, Dackor RT, Zeldin DC (2011). Cytochrome P450 2J2 is highly expressed in hematologic malignant diseases and promotes tumor cell growth. Journal of Pharmacology and Experimental Therapeutics.

[37] Wei X, Zhang D, Dou X, Niu N, Huang W, Bai J, Zhang GJ (2014). Elevated 14, 15-epoxyeicosatrienoic acid by increasing of cytochrome P450 2C8, 2C9 and 2J2 and decreasing of soluble epoxide hydrolase associated with aggressiveness of human breast cancer. BMC cancer.

[38] Zhou SF (2008). Drugs behave as substrates, inhibitors and inducers of human cytochrome P450 3A4. Current drug metabolism.

[39] Yasuda K, Ranade A, Venkataramanan R, Strom S, Chupka J, Ekins S, Schuetz E, Bachmann K (2008). A comprehensive *in vitro* and in silico analysis of antibiotics that activate pregnane X receptor and induce CYP3A4 in liver and intestine. Drug Metabolism and Disposition.

[40] Pfrunder A, Gutmann H, Beglinger C, Drewe J (2003). Gene expression of CYP3A4, ABC-transporters (MDR1 and MRP1-MRP5) and hPXR in three different human colon carcinoma cell lines. Journal of pharmacy and pharmacology.

[41] Dhaini HR, Thomas DG, Giordano TJ, Johnson TD, Biermann JS, Leu K, Hollenberg PF, Baker LH (2003). Cytochrome P450 CYP3A4/5 expression as a biomarker of outcome in osteosarcoma. Journal of clinical oncology.

[42] Nagaoka R, Iwasaki T, Rokutanda N, Takeshita A, Koibuchi Y, Horiguchi J, Shimokawa N, Iino Y, Morishita Y, Koibuchi N (2006). Tamoxifen activates CYP3A4 and MDR1 genes through steroid and xenobiotic receptor in breast cancer cells. Endocrine.

[43] Imaoka S, Hashizume T, Funae Y (2005). Localization of rat cytochrome P450 in various tissues and comparison of arachidonic acid metabolism by rat P450 with that by human P450 orthologs. Drug metabolism and pharmacokinetics.

[44] Nishimura M, Yaguti H, Yoshitsugu H, Naito S, Satoh T (2003). Tissue distribution of mRNA expression of human cytochrome P450 isoforms assessed by high-sensitivity real-time reverse transcription PCR. Yakugaku zasshi: Journal of the Pharmaceutical Society of Japan.

[45] Zeldin DC, Wei S, Falck JR, Hammock BD, Snapper JR, Capdevila JH (1995). Metabolism of epoxyeicosatrienoic acids by cytosolic epoxide hydrolase: substrate structural determinants of asymmetric catalysis. Archives of biochemistry and biophysics.

[46] Thompson CM, Capdevila JH, Strobel HW (2000). Recombinant cytochrome P450 2D18 metabolism of dopamine and arachidonic acid. Journal of Pharmacology and Experimental Therapeutics.

[47] Wang Y, Wei X, Xiao X, Hui R, Card JW, Carey MA, Wang DW, Zeldin DC (2005). Arachidonic acid epoxygenase metabolites stimulate endothelial cell growth and angiogenesis via mitogen-activated protein kinase and phosphatidylinositol 3-kinase/Akt signaling pathways. Journal of Pharmacology and Experimental Therapeutics.

[48] Cheranov SY, Karpurapu M, Wang D, Zhang B, Venema RC, Rao GN (2008). An essential role for SRC-activated STAT-3 in 14, 15-EET–induced VEGF expression and angiogenesis. Blood.

[49] Michaelis UR, Xia N, Barbosa-Sicard E, Falck JR, Fleming I (2008). Role of cytochrome P450 2C epoxygenases in hypoxia-induced cell migration and angiogenesis in retinal endothelial cells. Investigative ophthalmology & visual science.

[50] Yan G, Chen S, You B, Sun J (2008). Activation of sphingosine kinase-1 mediates induction of endothelial cell proliferation and angiogenesis by epoxyeicosatrienoic acids. Cardiovascular research.

[51] Luo J, Feng XX, Luo C, Wang Y, Li D, Shu Y, Wang SS, Qin J, Li YC, Zou JM, Tian DA, Zhang GM, Feng ZH (2016). 14,15-EET induces the infiltration and tumor-promoting function of neutrophils to trigger the growth of minimal dormant metastases. Oncotarget.

[52] Sander AL, Sommer K, Neumayer T, Fleming I, Marzi I, Barker JH, Frank J, Jakob H (2013). Soluble epoxide hydrolase disruption as therapeutic target for wound healing. J Surg Res.

[53] Namgoong GM, Khanal P, Cho HG, Lim SC, Oh YK, Kang BS, Shim JH, Yoo JC, Choi HS (2010). The prolyl isomerase Pin1 induces LC-3 expression and mediates tamoxifen resistance in breast cancer. Journal of Biological Chemistry.

[54] Khanal P, Yun H, Lim S, Ahn S, Yoon H, Kang K, Hong R, Choi H (2012). Proyl isomerase Pin1 facilitates ubiquitin-mediated degradation of cyclin-dependent kinase 10 to induce tamoxifen resistance in breast cancer cells. Oncogene.

[55] Matsuura I, Chiang KN, Lai CY, He D, Wang G, Ramkumar R, Uchida T, Ryo A, Lu K, Liu F (2010). Pin1 promotes transforming growth factor-beta-induced migration and invasion. J Biol Chem.

[56] Choi HK, Yang JW, Roh SH, Han CY, Kang KW (2007). Induction of multidrug resistance associated protein 2 in tamoxifen-resistant breast cancer cells. Endocrine-related cancer.

[57] Choi JM, Oh SJ, Lee SY, Im JH, Oh JM, Ryu CS, Kwak HC, Lee JY, Kang KW, Kim SK (2015). HepG2 cells as an *in vitro* model for evaluation of cytochrome P450 induction by xenobiotics. Arch Pharm Res.

[58] Kim ES, Kim JS, Kim SG, Hwang S, Lee CH, Moon A (2011). Sphingosine 1-phosphate regulates matrix metalloproteinase-9 expression and breast cell invasion through S1P3–Gαq coupling. Journal of cell science.

[59] Kim MS, Lee EJ, Kim HRC, Moon A (2003). p38 kinase is a key signaling molecule for H-Ras-induced cell motility and invasive phenotype in human breast epithelial cells. Cancer research.

[60] Auerbach R, Kubai L, Knighton D, Folkman J (1974). A simple procedure for the long-term cultivation of chicken embryos. Dev Biol.

[61] Colman R, Pixley R, Sainz I, Song J, IsordiaSalas I, Muhamed S, Powell J, Mousa S (2003). Inhibition of angiogenesis by antibody blocking the action of proangiogenic high-molecular-weight kininogen. Journal of Thrombosis and Haemostasis.

